# Bullous Variant of Sweet’s Syndrome as a Consequence of Radioiodine Contrast Exposure

**DOI:** 10.7759/cureus.3490

**Published:** 2018-10-24

**Authors:** Anusha Ganapati Bhat, Sudeep K Siddappa Malleshappa, Deepak Kumar Pasupula, Wayne Duke, Reham Shaaban

**Affiliations:** 1 Internal Medicine, Baystate Medical Center, Springfield, USA; 2 Internal Medicine, University of Pittsburgh Medical Center, Pittsburgh, USA; 3 Pathology, Baystate Medical Center, Springfield, USA; 4 Internal Medicine, Baystate Medical Center, Springfield , USA

**Keywords:** sweet's syndrome, bullous variant sweet syndrome, radioiodine contrast

## Abstract

Bullous variant of Sweet’s syndrome (SS) is a rare form of SS, which clinically presents as bullous hemorrhagic rash and demonstrates dermal neutrophilic infiltrates with segregation of dermo-epidermal junction histopathologically. We present a case of a 73-year-old patient, who initially developed a hypersensitivity reaction on exposure to a radiocontrast agent and subsequently developed blistering rashes, which were established to be from bullous SS after exclusion of other possible diagnoses. Contrast media are utilized commonly in the current era of medicine and SS is rarely identified as an adverse event from it. Bullous variant particularly presents aggressively, which when recognized early responds to steroid use with clinical recovery.

## Introduction

Radiological technologies are an integral part of the current day medicine that are widely being used for diagnostic and therapeutic purposes. Unfortunately, the use of contrast media is also associated with side effects. A rarer manifestation is that of acute febrile neutrophilic dermatosis, also called as Sweet’s syndrome (SS), which has been less recognized with contrast use [1-2]. Bullous variant is an aggressive form of SS mainly triggered from strong circulatory inflammatory cytokines which present as bullous hemorrhagic rash and demonstrate dense neutrophilic infiltrates in dermis with focal segregation of dermo-epidermal junction due to inflammatory damage. This particular variant has never been associated with contrast-related SS to the best of our knowledge and is necessary to be recognized early. SS responds successfully to steroids, but when not recognized or treated may lead to disastrous sequelae, including death.

## Case presentation

**Figure 1:**
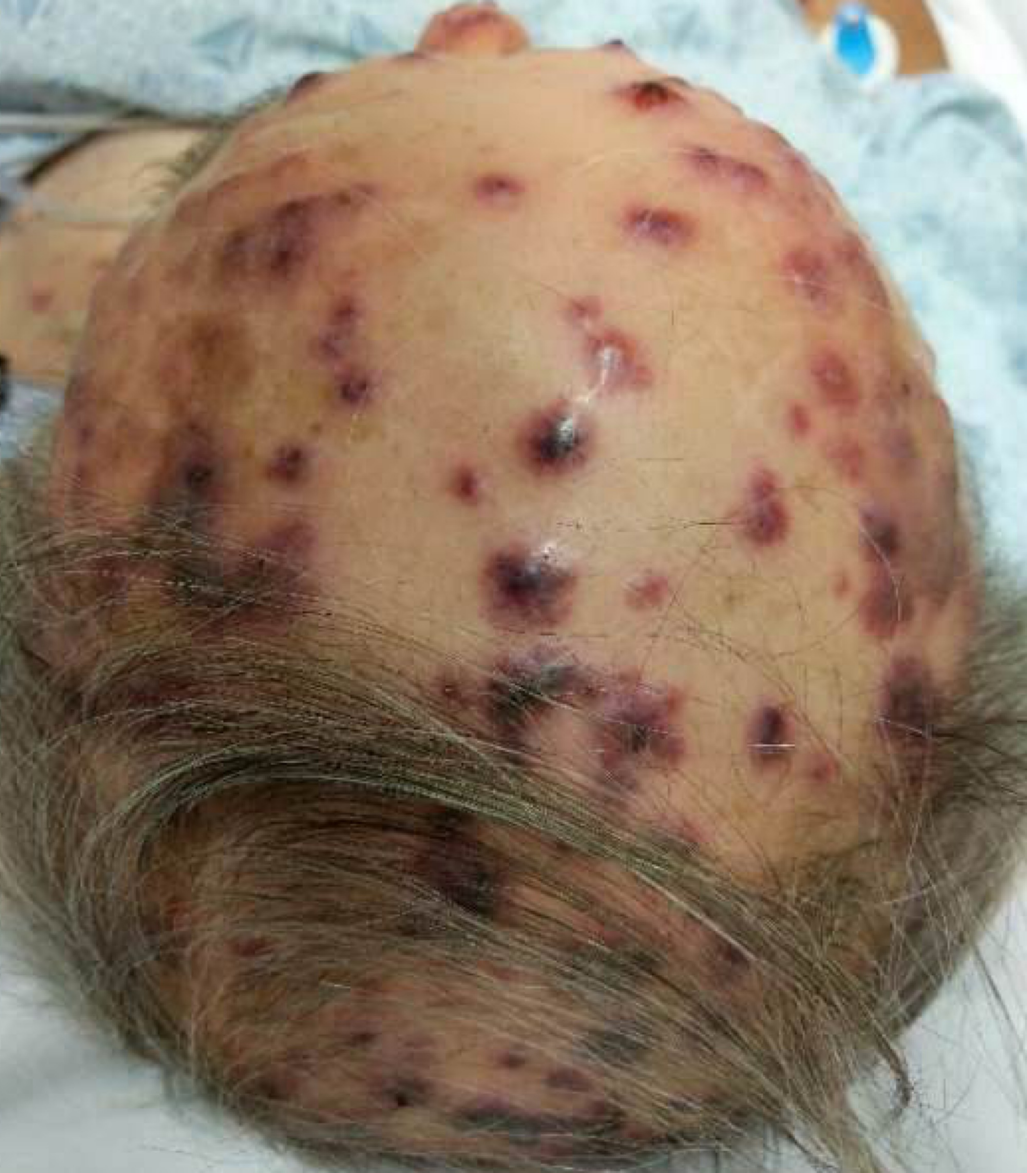
Hemorrhagic lesions on the scalp.

**Figure 2:**
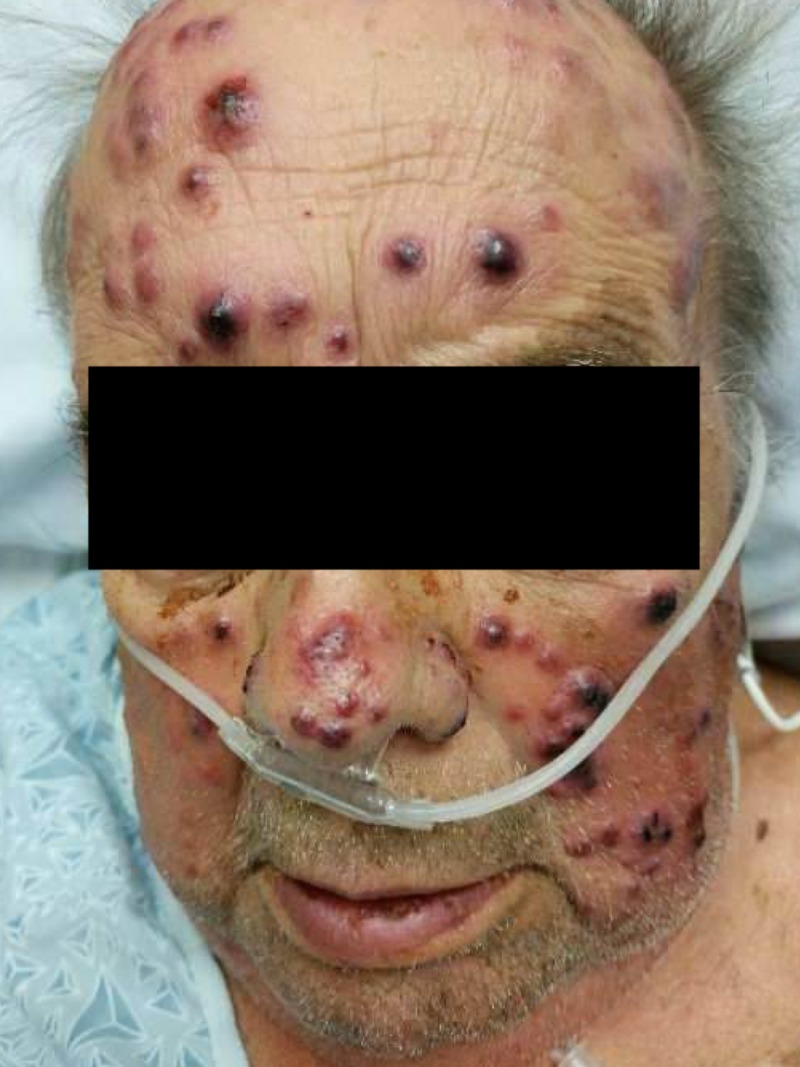
Lesions on the face.

**Figure 3:**
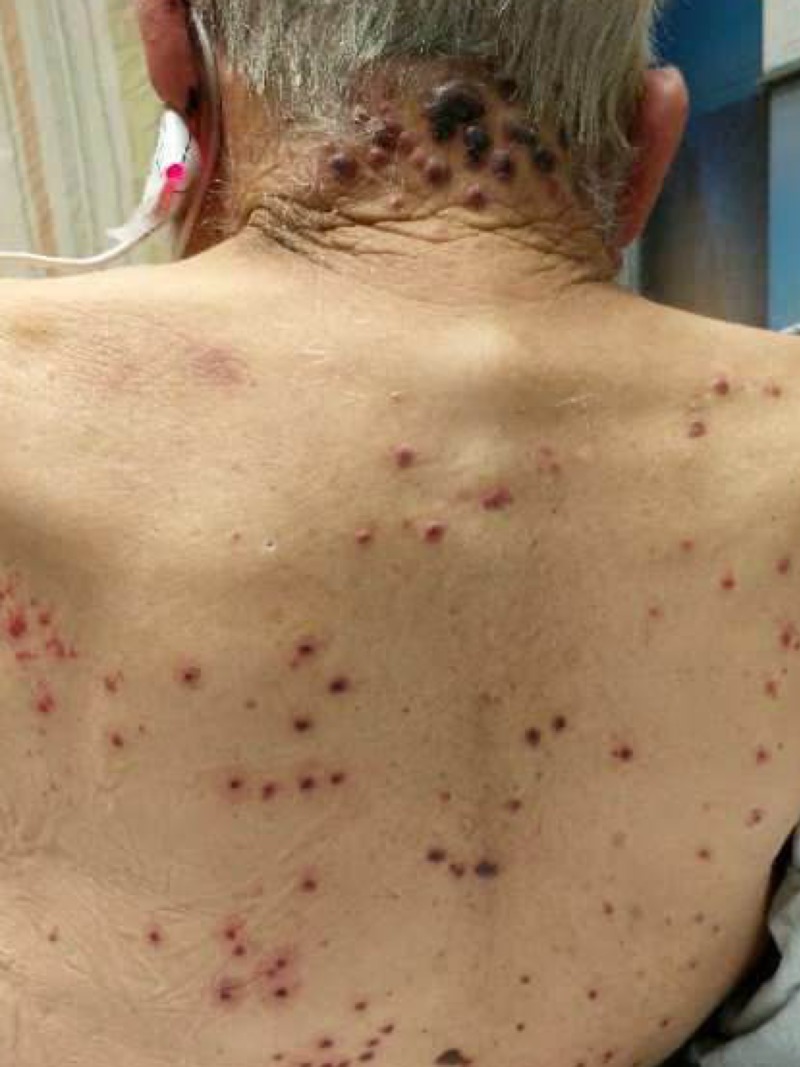
Bullous hemorrhagic lesions on the nape of the neck and back.

A 73-year-old male with a past history of antineutrophil cytoplasmic antibody (ANCA) vasculitis, and end-stage renal disease on hemodialysis presented with acute onset hemorrhagic lesions for a day. He had no prior allergies. Two days before the current presentation, he had undergone a computed tomography (CT) scan of the abdomen with intravenous radioiodine contrast for evaluation of an acute episode of abdominal pain. Soon after administration of the radioiodine contrast, he developed generalized hives which resolved with anti-histamines. However, over the next 24-hour period he developed bullous hemorrhagic rash which initially began at the nape of his neck and later centrifugally spread to his face, chest, and back (Figures 1-3). 

**Figure 4:**
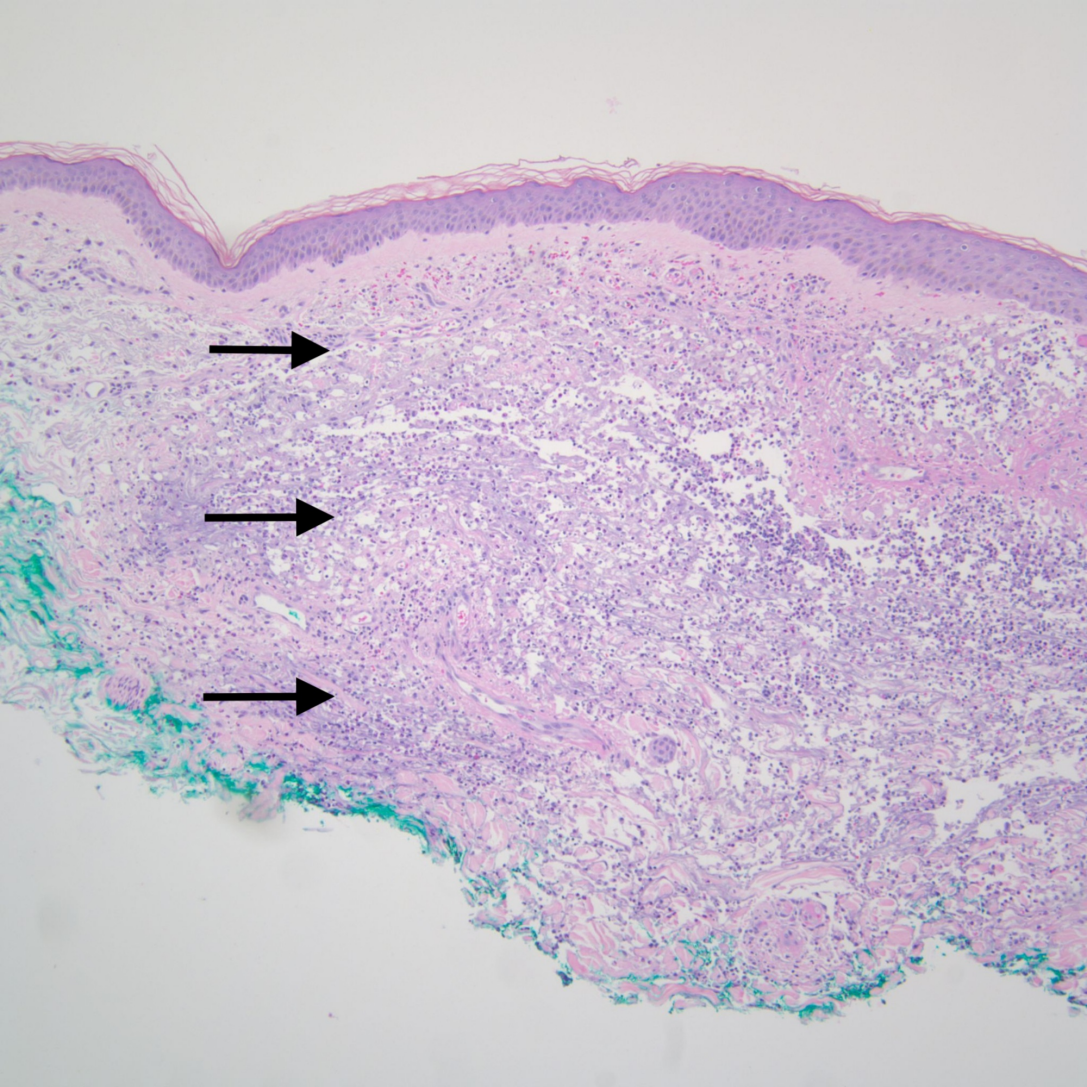
A 100X image of the skin biopsy with H&E stain showing a normal epidermis with underlying inflammatory infiltrates (arrows) spanning the papillary and reticular dermis. The infiltrates extend up to the base of the biopsy.

**Figure 5:**
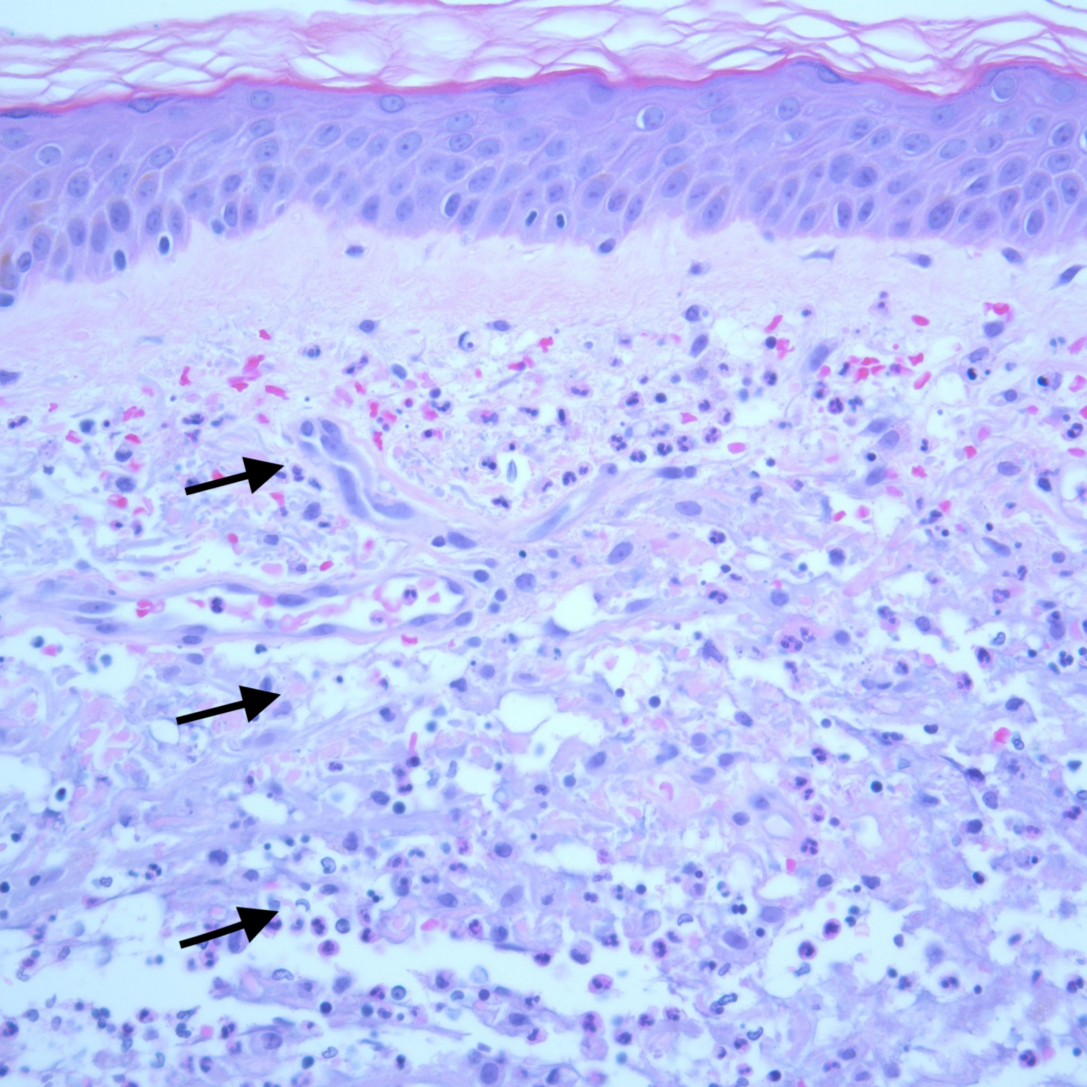
At 400X, the infiltrates (arrows) are seen to be composed of neutrophils admixed with leukocytoclasis and collagen degeneration. As stains for microorganisms were negative, the findings were interpreted to be consistent with Sweet’s syndrome.

Rashes were associated with fatigue, photophobia, and fever. On examination, he had an oral temperature of 101.1°F (normal = 97°F-99°F) with multiple well-demarcated tender hemorrhagic bullae and plaques. Laboratory workup was significant for leukocytosis of 12,000 per microliter of blood (normal = 4,000 and 11,000 per microliters of blood), chronic stable thrombocytopenia of 88,000 microliters of blood (normal = 150,000-450,000 platelets per microliter of blood), elevated sedimentation rate of 33 mm/hour (normal = 0-22 mm/hour for men), elevated C-reactive protein of 18 mg/dL (normal </= 3 mg/dL), and low complement C3. Due to a history of ANCA vasculitis, he was re-evaluated and found to have a positive perinuclear ANCA and >100 U myeloperoxidase antibody. Dermatology was involved and a shave biopsy of the skin lesion measuring 0.7 cm x 0.7 cm x 0.1 cm was obtained. On hematoxylin and eosin (H&E) stain, pathology was significant for neutrophils admixed with nuclear debris and collagen degeneration spanning throughout the dermis with focal degeneration and separation of epidermis from underlying papillary dermis (Figures 4-5). 

Infectious workup including blood cultures for bacteria, fungi, special stains for skin biopsy with Grocott’s methenamine silver stain, mucicarmine and immunochemistry for cryptococcus, herpes simplex, and bacteria were all negative. Thus, SS was established on clinical and histopathological basis. The patient was managed with high-dose prednisone course of 40 mg/day for a week with complete resolution of his skin manifestations. He had an uneventful recovery and was discharged home safely.

## Discussion

Iodinated radiocontrast media are widely used for over 70 million diagnostic tests across the world. The contrast helps to improve delineation of the tissue details in the body, but have been associated with higher rates of adverse events, particularly with hypersensitivity reactions as compared to those utilizing no contrasts [3]. One such reaction is of acute febrile neutrophilic dermatosis, previously known as Gomm-Button disease and currently familiar as SS. It is an inflammatory reaction of the skin triggered either by infection, autoimmune conditions, pregnancy, malignancy, or exposure to drugs. They are classified based on the etiology as classical SS, malignancy-associated SS, or drug-induced SS. 

Even though SS was first described in 1964 by Sir Robert Douglas Sweet, mainly in patients with recent infections, severe complicated inflammatory bowel disease or other idiopathic etiologies, temporal relation to drugs were not established until 1986 [4-5]. In 1986, Su and Liu established drug-induced SS from trimethoprim-sulfamethoxazole [4]. Ever since then, several medications, primarily granulocyte-colony stimulating factor (G-CSF) have been associated with drug-induced SS. In 1996, Walker and Cohen established criteria to classify SS to be drug related [6]. Drug-induced SS is characterized by the presence of all of the following clinical manifestations: temporal development of painful skin lesions following a drug exposure, fever of 38.4°C, histopathology of dense neutrophilic infiltration into the dermis without vasculitis, and resolution of symptoms following steroid use, which were all met in our case. 

The pathophysiology behind SS is of delayed hypersensitivity reaction, likely from T-cell mediated hypersensitivity, which develops within the few hours of radioiodine contrast administration [1-2]. Our case is unique for the fact that the patient also demonstrated skin blisters, which have not been previously described with radioiodine-induced SS. Marzano et al. studied inflammatory markers contributing to the development of skin lesions based on immunohistochemistry, where expression of inflammatory markers like CD3, CD163, tumor necrosis factor-alpha, interleukin-8, interleukin-17, matrix metalloproteinase-9, and myeloperoxidase were increased in SS and very aggressively expressed among the bullous variants, leading to pronounced neutrophilic activation, chemotaxis, and increased tissue breakdown when compared to papulo-nodular patterns [7]. These mechanisms may be hyperactivated in a patient with pre-existing inflammatory conditions like ANCA-vasculitis, as in our case. Once developed, the inflammation responds to oral steroids. Patients with severe relapsing disease may require longer taper of steroids. 

Thus, SS is an infrequently seen adverse event from radioiodine contrast agents, which may develop aggressively, particularly in patients with high-risk comorbidities like ANCA vasculitis, inflammatory bowel disease, infections, pregnancy and malignancies, which have been established to trigger SS. 

## Conclusions

We report a case of an elderly male with a history of ANCA vasculitis, who developed acute hemorrhagic bullae within 24 hours of radioiodine contrast administration. Lesions were established to be bullous SS, an aggressive form of SS, which resolved with oral steroid use. Thus, recognizing the population at risk for development of SS and watching for any erupting symptoms during the initial timeframe following administration of contrast media can lead to early recognition and prevention of unfavorable outcomes.
